# The Effect of Group Attachment and Social Position on Prosocial Behavior. Evidence from Lab-in-the-Field Experiments

**DOI:** 10.1371/journal.pone.0058750

**Published:** 2013-03-26

**Authors:** Delia Baldassarri, Guy Grossman

**Affiliations:** 1 Sociology Department, New York University, New York, New York, United States of America; 2 Political Science Department, University of Pennsylvania, Philadelphia, Pennsylvania, United States of America; Universidad Carlos III de Madrid, Spain

## Abstract

Social life is regulated by norms of fairness that constrain selfish behavior. While a substantial body of scholarship on prosocial behavior has provided evidence of such norms, large inter- and intra-personal variation in prosocial behavior still needs to be explained. The article identifies two social-structural dimensions along which people's generosity varies systematically: group attachment and social position. We conducted lab-in-the-field experiments involving 2,597 members of producer organizations in rural Uganda. Using different variants of the dictator game, we demonstrate that group attachment positively affects prosocial behavior, and that this effect is not simply the by-product of the degree of proximity between individuals. Second, we show that occupying a formal position in an organization or community leads to greater generosity toward in-group members. Taken together, our findings show that prosocial behavior is not an invariant social trait; rather, it varies according to individuals' relative position in the social structure.

## Introduction

Social organization affects prosocial behavior [1]–[7]. Although some people are more other-regarding than others, most individuals are neither universally altruistic nor selfish. For example, when asked to allocate resources between themselves and a stranger in a dictator game (DG), individuals share, on average, between 20 to 30% of their endowment. However, inter-personal variation is quite large: the modal behavior is, in fact, to give nothing, while a few give up to a half or more [8], [9]. Moreover, varying the identity of the recipient or the conditions of anonymity leads to significant intra-personal variation: individuals do not only share larger amounts of resources with their kin [10], but are also more likely to share resources with friends and acquaintances than with strangers [11]–[13]. Shared identities, such as ethnicity [14], [15], religion [16], or political partisanship [17], have also been shown to affect individuals' other-regarding preferences.

It is widely accepted that social life is regulated, to some extent, by distributive and reciprocity norms that constrain selfish behavior. However, we still lack a comprehensive understanding of inter- and intra-personal variation in prosocial behavior [18]. While socio-demographic characteristics (such as wealth, education and age) are neither powerful nor stable predictors of prosocial behavior [8], [11], [19], [20], there is some evidence suggesting that other-regarding preferences are affected by structural factors, such as a society's level of market integration [21] or its patterns of social relationships [22]–[24]. To the extent that social structure has been an object of investigation, it has been largely studied using a social network conceptualization [11]–[13], [25]–[29], and analyses are often based on the notions of *network proximity* and *network centrality*.

In this article we identify two major sources of variation in prosocial behavior, both related to individuals' relative position in the social structure: group attachment and formal position. Complementing the social networks approach, which is based on ‘tangible’ interpersonal relations, our mechanisms of group identification and occupancy of formal roles rely on processes of categorization and generalization that induce individuals to extend their prosocial behavior beyond the immediate circle of kin and acquaintances.

In small groups and very cohesive societies, close-knit networks can be sufficient to support high levels of cooperation through mechanisms of kin selection, reciprocal altruism and costly signaling [20], [29]. However, as group size and societal complexity increase, it is imperative that constraints on selfish behavior become less dependent on direct interpersonal relationships [2], [30]. In their seminal work on fairness in 15 diverse societies, Henrich et al. [31] explain the greater levels of prosocial behavior documented in large-scale, market integrated societies as stemming from an evolutionary process in which prosocial norms, such as generalized trust, emerge “to sustain mutually beneficial exchanges in contexts where established social relationships were insufficient” ([31], p. 1480). According to social identity theory, individuals rely on categorization schemas, which allow them to generalize their interpersonal experiences to a broader class of alters and to relate to others even in the absence of a personal (direct or indirect) relationship. Unfamiliar others are, thus, instantaneously classified as members of in- or out-groups based on certain distinctive traits – e.g., ethnicity, gender or class – that are relevant in a given social context [32]. Once this distinction is made, the strength of group-specific identity and sense of belonging helps to determine the extent to which individuals consider the preferences of alters who have been classified as group members [33]. This process is at the basis of our *group attachment* hypothesis.

The process of social differentiation that characterizes complex societies brings with it a second form of categorization and generalization: namely, that high status individuals often acquire formal roles (i.e., tribal chief, group leader, mayor, officer, etc.) that confer authority upon them. A key aspect of this form of social differentiation is that it goes hand-in-hand with role-specific expectations that transcend the identity of the person occupying the formal role [34]–[37]. Acting on the basis of these expectations, leaders may feel a stronger obligation to consider the preferences of group members, or they may have a stronger incentive to share resources in order to signal ability or competence [18]. Finally, in societies in which patron – client relations are ubiquitous, sociological and anthropological accounts suggest that leaders exhibit prosocial behavior to send credible signals that they command resources that loyal followers may be able to enjoy [38]. Under such conditions, excessive sharing by office holders may be a strategy designed to increase one's influence [39], build alliances and secure political support [40]. In all cases, the observed outcome would be an increase in prosocial behavior as a function of formal leadership roles. We refer to this process as the *formal position* hypothesis.

In this paper we offer empirical evidence in support of both the group attachment and formal position hypotheses, thus moving forward our understanding of the socio-structural factors that bring about prosocial behavior in complex societies.

### Proximity, Group Attachment, and Prosocial Behavior

Recent studies have demonstrated that generosity increases as the *social distance* between a giver (ego) and a receiver (alter) diminishes, but fail to distinguish between two separate components of social distance: (a) the *proximity* between individuals – which is related to ego and alters' frequency of interaction, to the amount of information ego has about alter, and to the nature of their personal relationship [41] – and (b) *group attachment*, which derives from the strength of their abstract identification with members of a group.

Adopting a *social network conception* of social distance, a first group of studies has shown that individuals are most willing to share their resources with people they are directly connected to, as well as to exhibit greater prosocial behavior towards people who are just a few step removed in their social network (e.g., friends of friends) than toward more distant others [11], [12], [25]. Social distance in this framework is measured using the geodesic distance between ego and alter, thus inevitably conflating network proximity with the content and strength of relationships. In addition, social network studies of prosocial behavior are often based on closed systems – e.g., high-school students [11], hunter-gatherer societies [25] or small villages [12] – and rely on an exclusive set of relationships in which individuals are embedded. Individuals in modern societies, however, belong to multiple and non-overlapping groups [42] and their identifications and allegiances may vary from group to group by individual or collective experience. While we expect ‘tangible’ relationships to impact generosity even in contexts where individuals are embedded in multiple, overlapping networks, the level of prosocial behavior we observe in more complex social systems cannot be accounted for exclusively on the basis of interpersonal relationships [1], [2], [4].

*Social identity theory* complements the network approach to explain variation in other-regarding preferences. Adopting an identity-based conception of social distance, a few studies have demonstrated that individuals are more willing to share resources with in-group than with out-group members. In-group favoritism has been observed not only where group membership was based on ascribed categories, such as, ethnicity [14], [15], religion [16], or political partisanship [17], but also in cases where it was randomly assigned [43], as well as in laboratory settings where scholars induced ‘minimal’ or trivial group identities [32], [44], [45].

The foundation of social identity theory is that a person's sense of self is derived from his or her membership in social groups. Once a person identifies herself as part of a group, she is likely to behave in prescribed or expected ways toward members of that group [32], [46] Group identification results from a process of categorization, identification, and comparison in which individuals (including oneself) are classified into groups by context-specific attributes. Ego's prosocial behavior toward a person classified as an in-group member does not (necessarily) stem from their proximity; rather, it is (at least partially) derived from the ego's level of *attachment* to his or her shared group.

Analytically, it is important to note that group attachment is a dispositional mechanism that transcends proximity to and knowledge of a specific member of the group. We define group attachment as the strength of one's identification with a group, and we hypothesize that the stronger one's identification, the more willing he or she will be to share resources with group members. Though both *social proximity* and *group attachment* lead to in-group favoritism, the reasons behind this observed behavior are rather different. Proximity is based on particularized past experiences, while group attachment can be viewed as a generalization from past experiences, extending them to a broader set of people. Exposure to a subset of in-group members fosters positive expectations about the group in general, leading one to perceive its members as more honest, friendly, and trustworthy than members of out-groups [47], [48].

Empirically, however, social identity theory studies – like social network studies – are unable to isolate the effect of group attachment from that of social proximity: both mechanisms are simultaneously at work in most group experiences. First, group identification often originates from interaction with group members: people are likely to be more familiar with in-group members, and have more information about their needs, deeds and priorities. For instance, in their seminal study of cooperation in randomly assigned army platoons, Goette et al. base their argument on a group attachment mechanism while at the same time claiming that group identity was fostered during four weeks of intense training in which “officers interact [ed] almost exclusively with members of their own platoon” ([43], p. 213).

This paper is specifically designed to address some of the drawbacks of both social network and social identity theory. First it seeks to overcome the identification problem mentioned above, providing, to our knowledge, the first experimental evidence of the independent effect of group attachment on prosocial behavior. We do so by comparing variation in prosocial behavior as a function of *membership in overlapping groups*, holding proximity constant across groups. Secondly, we mitigate the problem of weak external validity by conducting a series of lab-in-the-field experiments in rural Uganda with members of pre-existing groups [49], [50].

Each of our study's subjects is a member of both a local coffee-producer organization in rural Uganda as well as a member of the village in which he or she resides (and often was born). Key for our identification is the fact that the level of proximity to members of both groups is either equal, or closer in the case of co-villagers. This is because the political, economic and social life of farmers in rural Uganda revolves around their village. Indeed, beside their interactions with immediate family members, the largest part of everyday interaction takes place with co-villagers. Villagers in our sample attend, on average, three community meetings a year, and are members of five community-level organizations. Villagers also do not spend much of their time outside the village: only a small fraction of our subjects attained a level of education that required traveling beyond the village, and only 15% of them report having a motorcycle in the household. In fact, almost all men in our sample were born in their village, while almost 3/4 of sampled women moved to their husband's village, typically in early adulthood.

For several reasons, however, we expect our study's participants to exhibit a stronger attachment to their farmer group than to their village. First, the farmer organization plays a central role in shaping farmers' welfare. Second, whereas membership in the village is, to a large degree, ascribed, joining one's farmer group takes place on a voluntaristic basis. Third, farmer group members share common characteristics: size of their land holdings, income, age, etc. and this homophily may favor group identification [51]. Using a dictator game to measure subjects' other-regarding preferences towards anonymous members of both the village and the farmer group, the first major contribution of our research is to show that group attachment strongly affects prosocial behavior, above and beyond the level of social proximity.

### Formal and Informal Social Position

Social organization does not only produce differentiation between groups, but also within groups, organizing individuals in characteristic network topologies and leading them to occupy different positions in the social hierarchy [25], [34]. While the specific network topology does not seem to affect overall levels of cooperation [52], there are at least three mechanisms that would induce one's *social position* in a group to impact his or her level of prosocial behavior toward its members. First, a high status position could bring with it greater expectations for prosocial behavior, and there may be reputation costs for not meeting such expectations. Closely related, generous behavior signals not only competence but also the intent to engage in beneficial exchange relations with those targeted by the leader's largess [18], [39], [40]. Finally, selection processes may lead people that exhibit a high degree of other-regarding preferences to occupy central positions in the social structure [53].

Scholars have so far used network centrality measures to capture individuals' social standing within groups, reporting results that are rather mixed. On one hand, several recent studies found that measures of network centrality do not correlate with more prosocial behavior in diverse contexts, such as indigenous tribes in Tanzania [25], Amazon's Mechanical Turk subject pool [52], and student volunteers in experimental laboratory settings [26], [27]. On the other hand, three recent studies found a positive correlation between network centrality and prosocial behavior, at least for some types of networks [13], [28], [39]. A clear limitation of several past studies is their reliance on a single network, thus basing their empirics on a single case-group.

This paper expands the scope of the emerging research on social position in several important ways. First we draw evidence from 50 different community organizations, which allows us to better test our hypotheses, especially those concerning social network position, across a large number of independent networks. Secondly, we examine a core aspect of social standing, hitherto overlooked: the *formal role* individuals occupy in a group. The second major contribution of our research is to show that individuals who occupy formal leadership positions in a group are more generous toward members of that group.

## Experimental Design

Lab-in-the-field experiments constitute a valuable complement to laboratory experiments when the goal is to study the effects of social organization on behavior [31], [34], [54]–[57]. To study processes in which group attachment and individual social position are expected to be consequential, we carried out lab-in-the-field experiments with a random sample of members of 50 different farmer cooperatives in rural Uganda. Each of these groups includes, on average, farmers from a cluster of 10 nearby villages. In each village, only a subset of residents chooses to join the local farmer group. Thus, by design, each of our subjects belongs to both a farmer cooperative and a village, and the overlap between these two groups is minimal. Each of our subjects may also occupy a formal position of authority within the farmer group (i.e., representatives in the cooperative's council or member of the cooperative's executive committee) and within the village (i.e., member of the village council). A total of 2,597 subjects participated in our experiment (see Table S1 in File S1 for descriptive statistics).

In each of the 50 different locations, participants were randomly assigned to one of two variants of a basic dictator game (DG). In a DG, two anonymous players are allotted a certain endowment – commonly 10 monetary units (MUs). The first player (the ‘decider’) has to allocate this sum between himself and the second player (the ‘recipient’). In this one-shot game, a purely self-interested ‘decider’ would give nothing to the second player. Thus, the amount contributed to the recipient in a DG is commonly interpreted as a measure of prosocial behavior, as participants decide under conditions of anonymity and are not at risk of sanctioning or losing their reputation [8]. Though it is common to interpret behavior in a DG as an expression of other-regarding preferences (pure altruism or generosity), some hold that it is more likely to be influenced by social norms, and are therefore agnostic with respect to whether people genuinely carry those preferences or instead simply follow prescribed norms [58]. Our conception of prosocial behavior encompasses both possibilities, without adjudicating between them.

In both variants of our experiment, subjects were invited to divide *two endowments* between themselves and two *different recipients* whose identities were unknown to the givers. In both experimental conditions, subjects were asked to divide one endowment between themselves and a *stranger*, defined as “a person selected at random from the sub-county”. We interpret this contribution as a baseline measure of respondents' generalized generosity (i.e., generosity toward a generalized other).

**Figure 1:**
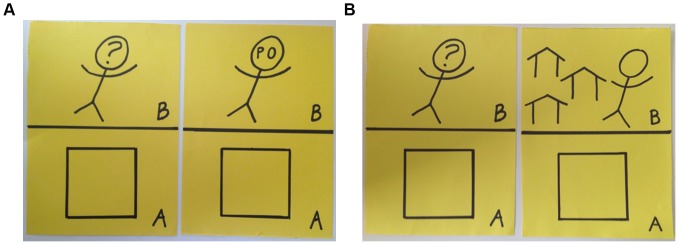
DG game boards for the PO member condition and co-villager condition. In both treatment conditions participants allocated one endowment between themselves and a stranger (identified by the question mark). The identity of the second recipient, however, varied across experimental conditions.

The identity of the second recipient, however, varied across experimental conditions. Subjects who were randomly assigned to the ‘villager’ condition were asked to divide the second endowment between themselves and a co-villager. Subjects who were randomly assigned to the ‘PO member’ condition were asked to divide the second endowment with a member of their producer organization. This contribution provides a measure of respondent's generosity towards members of a specific in-group (see section 3 in File S1 for the experimental scripts). The order in which subjects were confronted with the two choices was also randomized to eliminate sequence effects. Figure 1 shows the game boards for the two variants of the DG. Overall, this design allows us to test basic hypotheses regarding the effect of social distance and formal social position on prosocial behavior: first, we confirm findings concerning the overall effect of social distance; second, we isolate the effect of group attachment; and third, we document the relationship between formal leadership positions and in-group generosity.

## Results

### Social Distance and Group Attachment

First, we show that DG contributions increase as the social distance between decider and recipient decreases: on average, participants give a larger share of their endowment to in-group members (whether co-villagers or co-farmer group members) than to out-group members (‘strangers’). Analyzing *within-person variation*, we find that experimental subjects contribute, on average, 2.84 MUs to strangers, whereas they contribute a significantly larger share of their endowment to co-villagers (3.07 MUs, p-value  = 0.002). Similarly, the experimental subjects share a significantly larger share of their endowment with farmer group co-members than with strangers (4.07 MUs, p-value  = 0.000).

**Figure 2:**
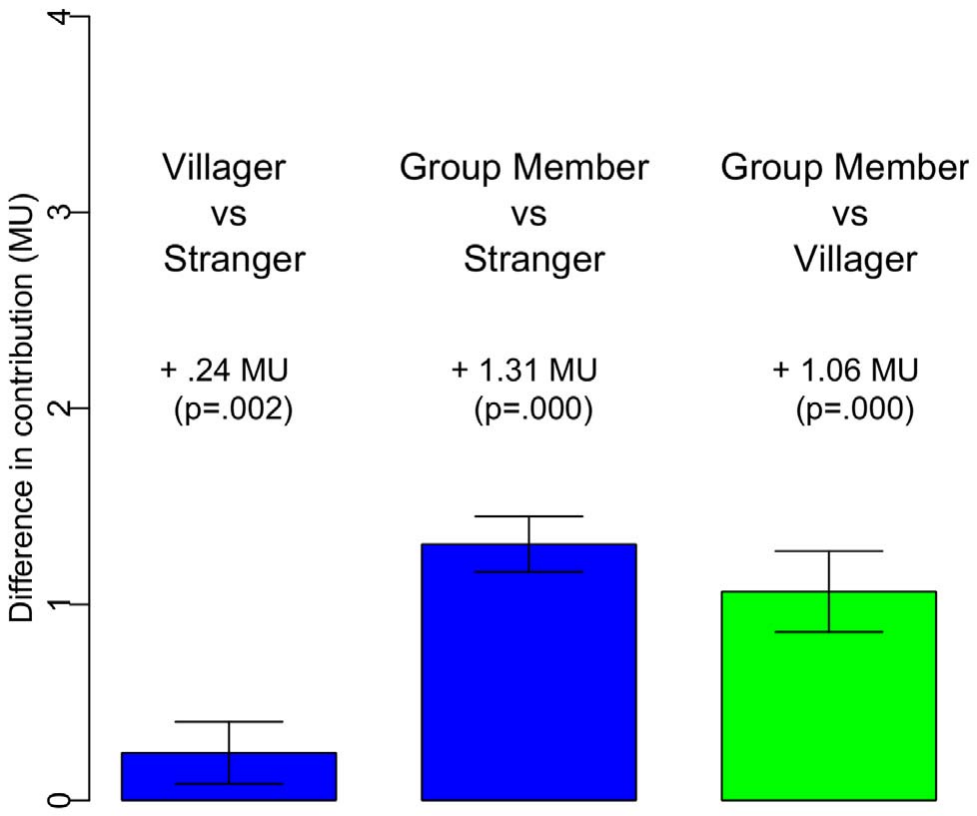
Treatment effect of the two variants of the dictator game. Participants allocated 0.24 MUs more to co-villagers and 1.31 MUs more to farmer group co-members (blue bars) than to ‘strangers’. Importantly, participants allocated 1.06 MUs more to farmer group co-members than to co-villagers (green bar). N = 2421. Whiskers indicate the 95% interval.

Second, we demonstrate that other-regarding preferences greatly vary between the two in-groups. To analyze *between-person variation* in contributions, we run a series of multilevel regressions in which the dependent variable is the difference between the contribution to in-group members and strangers and the key independent variable is the treatment variant (‘co-villager’ or ‘farmer group member’). Results are presented in Table 1: in Model I we report mean differences, whereas in Model II we also control for overall levels of altruism, as measured by subjects' contribution to the stranger. In Model III we add individual-level controls (male, age, education, wealth, church attendance, and being born in the village). Figure 2 reports the predicted differences in contribution, and 95% confidence intervals.

**Table 1:** Treatment effect of the two variants of the dictator game.

	Model I		Model II		Model III	
	*β*	st. err.	*β*	st. err.	*β*	st. err.
Group-member vs. Villager (ATE)	1.060***	(0.116)	1.064***	(0.103)	1.063***	(0.105)
Stranger contribution			−0.545***	(0.017)	−0.545***	(0.018)
Male					0.137	(0.081)
Age (units of 10)					−0.021	(0.027)
Church attendance					0.050	(0.066)
Education (standardized)					−0.022	(0.037)
Wealth (standardized)					−0.002	(0.037)
Intercept	.232***	(0.086)	1.777***	(0.092)	1.639***	(0.255)
√Ψ_(*a*)_	−19.943***	(3.803)	−2.476	(0.065)	−2.280	(1.479)
√Ψ_(*b*)_	0.859***	(0.057)	−0.234***	(0.065)	−0.235***	(0.067)
σ*_e_*	1.857***	(0.028)	0.451***	(0.015)	0.457***	(0.016)
N	2591		2591		2421	
Log Likelihood	−5449.15		−5034.80		−4721.23	

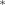
 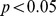 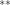 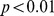 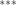 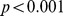.

The dependent variable is the difference in the contribution to stranger and to in-group member (farmer group member or co-villager). Results derived from a series of three-level random intercept linear regression models in which individuals are nested within farmer associations and interviewers, to control for group and interviewer effects. 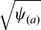 refers to variability between farmer groups, 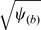 refers to between interviewers variability, and 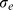 is the estimated standard deviation of the overall error term.

As mentioned, key to our research design is the fact that the experiment's subjects share the same level of social proximity to members of the two in-groups. That they choose to allocate 1.06 MUs more to an anonymous member of their farmer group than to an anonymous co-villager (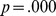), provides strong evidence that group attachment, and not only social proximity, impact prosocial behavior. Finally, it is worth noting that the subjects' personal characteristics (sex, age, income etc.) are not significant at conventional levels, either separately or jointly. The F-test for joint significance is 0.585.

### Formal Status and Prosocial Behavior

Our hypothesis regarding the effect of group attachment focuses on the way in which group identification impacts the relationship between a giver and different receivers. By contrast, our hypothesis on the effect of *social position* concerns the extent to which formal and informal positions that a subject occupies in the group structure have a bearing on his or her willingness to share resources with group members. First, we test this hypothesis by examining whether people who hold a formal leadership position in a group allocate a larger share of their endowment to members of that group. Here we rely on the fact that both the farmer group and the village hold periodic elections for various leadership positions.

**Figure 3:**
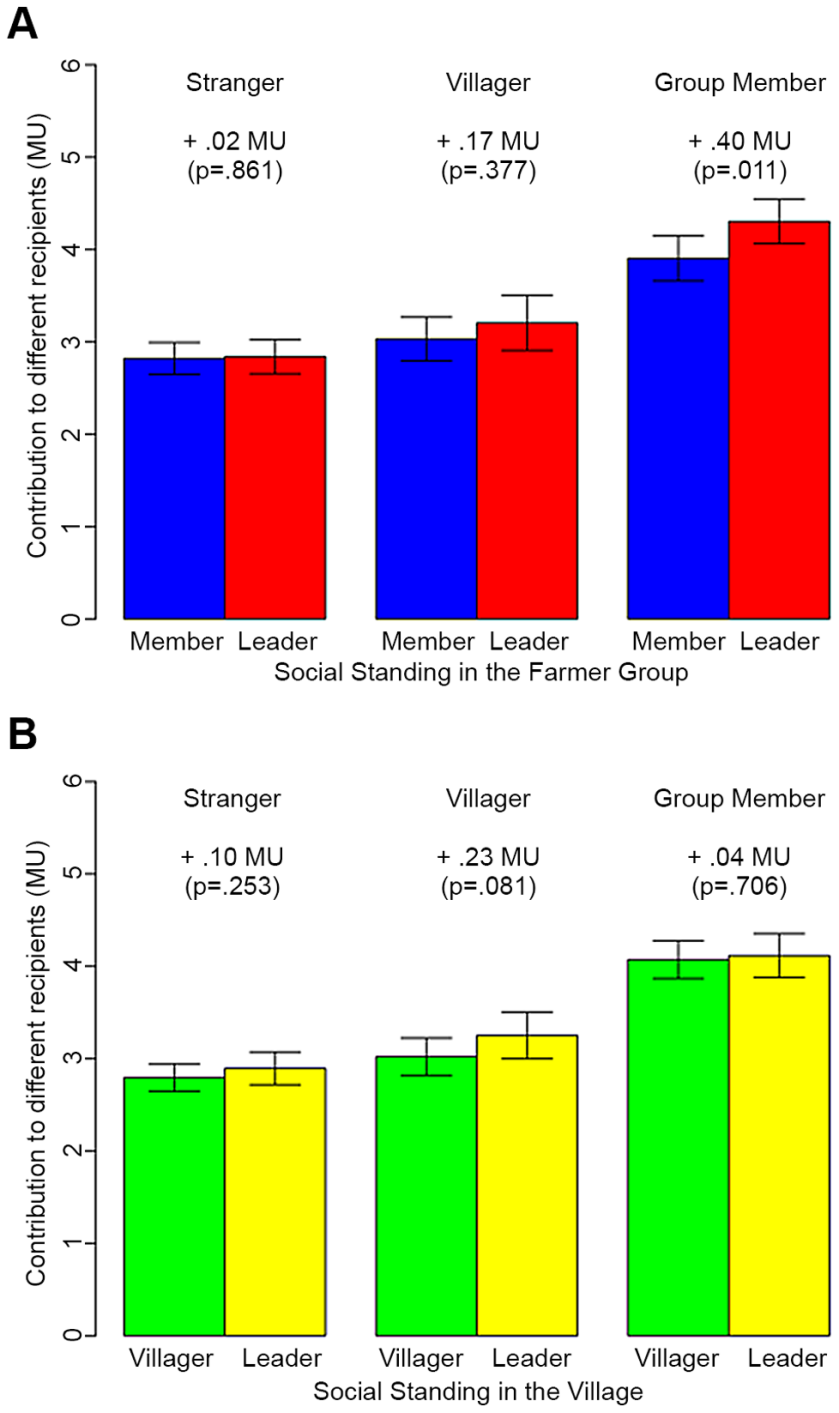
Holding a formal position increases prosocial behavior toward in-group members. Farmer group leaders allocated to members, on average, 0.40 s MU more than regular farmer-group members (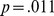). Leaders in the village gave, on average, 0.23 MUs more to co-villagers than regular villagers (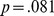). The contribution of both types of leaders did not differ from average group members when receivers were either strangers or members of a group in which they did not hold formal leadership positions. Predicted average contributions derived from multilevel regressions in which we model the contribution to strangers, villagers and farmer group members, controlling for individual and group level characteristics. Whiskers indicate the 95% interval, while the difference between regular members/villagers and leaders and its significance is reported in the graph. See SI Appendix, Table 1 for results in tabular form.

Figure 3 reports the predicted average contribution to strangers, co-villagers, and farmer group members. (To examine the relationship between formal leadership position and prosocial behavior we run a set of multilevel regression models in which the dependent variable is the contribution to strangers, villagers, and farmer group members respectively, and the independent variables of interest are indicators of whether individuals are village leaders and/or farmer group leaders. In all regressions we also control for individual and group level characteristics, as in the previous regression models. For results in tabular form see also Table S2 in File S1.) In the top panel, we distinguish between leaders and regular members of the farmer cooperative, and in the bottom panel, between village leaders and ‘regular’ villagers. Our first finding is that in both groups – the farmer group and the village – we do not find differences between leaders and regular members with respect to their contribution to strangers. This finding suggests that baseline generalized generosity is unaffected by formal leadership position. Second, in both groups we find that compared to regular members, leaders allocate significantly more to anonymous in-group members. For example, those holding formal leadership positions in the farmer cooperative gave, on average, 0.40 MUs more than regular members to other members of the farmer group (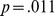). Similarly, village leaders gave, on average, 0.23 MUs more to co-villagers than regular villagers, though this difference is significant only at the 90% confidence interval (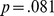).

Putting the two findings together, we conclude that leaders' greater willingness to share resources with in-group members applies only to members of the group in which they occupy a leadership position. Farmer group leaders do not share a larger part of the initial endowment with co-villagers when compared to average farmer group members. Similarly, village leaders do not share a larger part of the endowment with farmer group members than regular villagers do. This suggests that formal social position in a group induces greater prosocial behavior targeted specifically at members of that group (and that group only).

### Informal Status and Prosocial Behavior

Focusing on a subset of our subjects – farmers serving on their cooperative's council – we were also able to test whether *informal* network position in a group structure is related to greater prosocial behavior toward group members. In each of the 50 farmer organizations, we collected complete network information on the friendship, communication, and advice relationships among members of the farmer association council (see Materials and Methods section for further details).

As an indicator of informal social standing, for each of the networks we computed three centrality measures: degree, betweenness and eigenvector centrality [59], [60]. Confirming previous scholarship [11], [25]–[27], [52], we do not find a significant correlation between any measure of network centrality and subjects' level of generosity, as measured in the DGs (see Table S3 in File S1).

**Figure 4:**
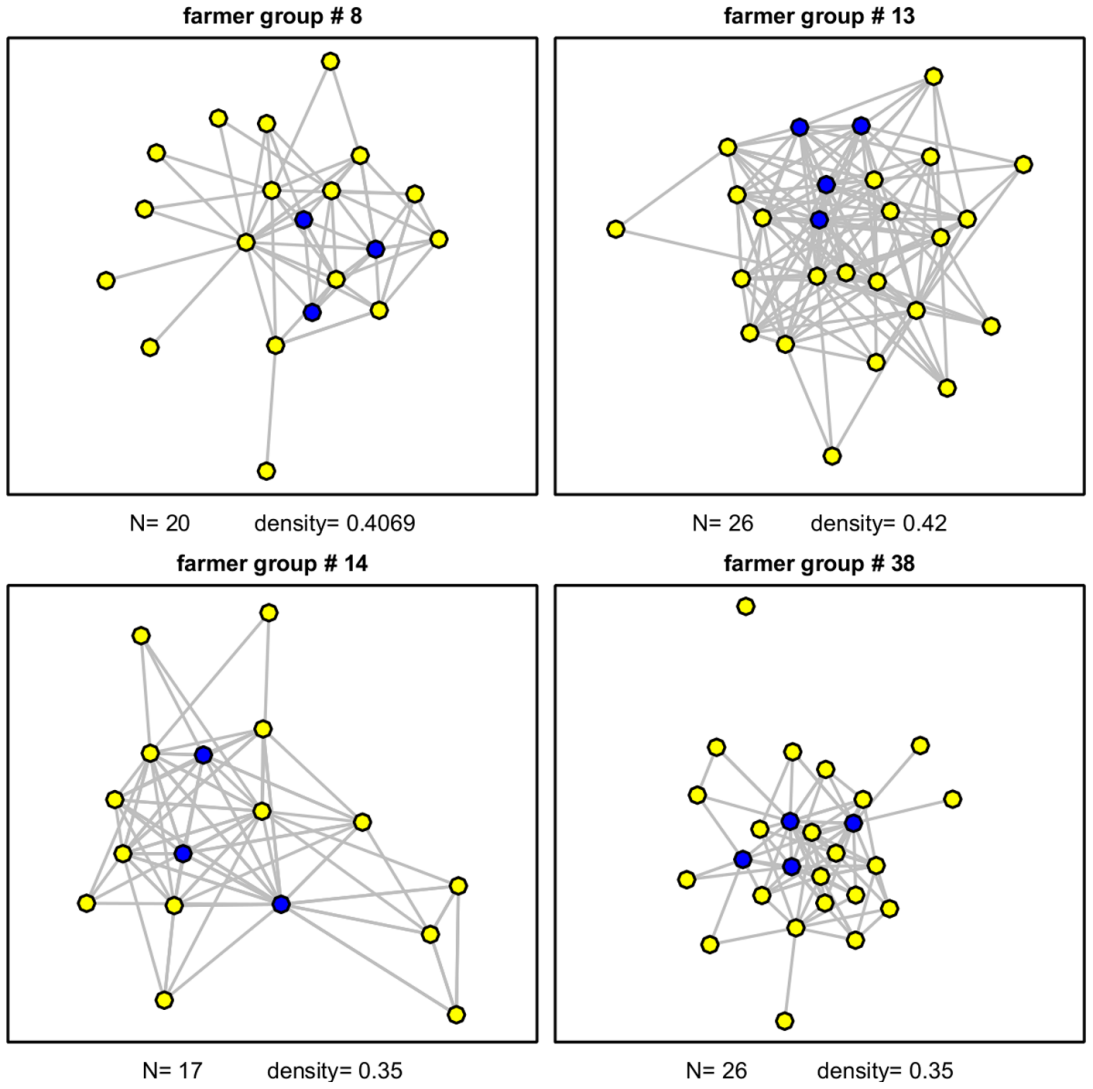
Relationship between formal and network position in the social structure. High-rank leaders (blue nodes) occupy network positions of greater centrality. The graphs show the structure of the communication network of four randomly selected farmer groups in our sample.

In the last part of this analysis, we examine the relationship between informal social standing and formal leadership position. We find that the most important leaders (members of the executive committee: e.g., the manager, the secretary and the treasurer of the farmer association) are more likely to occupy central positions in the leadership network. To illustrate this aspect, Fig. 4 visualizes the communication network for four farmer groups in our sample: in each graph, group executives (blue nodes) tend to occupy more central positions than village-level representatives (yellow nodes).

Systematic evidence in support of this finding is presented in Table 2, where we report average levels of network centrality measures for both representatives and members of the executive committee. In all three types of networks – friendship, communication and advice – members of the DC executive committee have significantly higher centrality measures than village representatives. Taken together, these findings suggest that while informal position within a group structure is positively correlated with the likelihood of occupying a formal position in the network, prosocial behavior is, nonetheless, more closely related to a formal position than to an informal one.

**Table 2:** Network centrality by leadership position.

	Position		
	(Reps)	(DC Exec)	p-value
degree friend	20.34	25.67	0.000
	(13.46)	(14.90)	
degree communication	29.80	33.16	0.001
	(12.81)	(14.60)	
degree advice	20.14	29.14	0.000
	(12.23)	(15.31)	
betweenness friend	15.57	38.50	0.000
	(21.24)	(51.55)	
betweenness communication	11.22	19.82	0.000
	(13.95)	(27.92)	
betweenness advice	11.54	61.18	0.000
	(20.40)	(112.9)	
eigenvector friend	0.564	0.769	0.000
	(0.234)	(0.209)	
eigenvector communication	0.692	0.829	0.000
	(0.189)	(0.184)	
eigenvector advice	0.536	0.801	0.000
	(0.202)	(0.178)	
Observations	855	168	

p-values derived from OLS models, in which each network centrality measure is regressed on an indicator measure of leadership position (whether village representative (0) or member of the executive committee (1). Standard errors are clustered at the farmer association level in parentheses.

### Social Position: Possible Explanatory Mechanisms

We now examine some of the reasons group members holding formal leadership positions might share a larger part of their endowment with other group members. We can safely exclude two alternative hypotheses. First, since leaders do not give more to strangers, we exclude the possibility that altruistic people are more likely to be selected for leadership positions. Second, since social standing in a group induces greater prosocial behavior targeted specifically at members of that group, and that group alone, we exclude the possibility that leaders are more sensitive to social distance considerations. We provide evidence, instead, that leaders' prosocial behavior, at least as measured by the DGs, may reflect the fact that community leaders are internalizing greater expectations since individuals hold their leaders to higher standards of fairness.

To explicitly test this mechanism, we conducted a series of Ultimatum Game (UG) experiments. In a UG a pair of players are allocated an amount of money (commonly 10 monetary units). The first player, the ‘decider’, is responsible for dividing this initial endowment with a second player, the ‘receiver’. The receiver then has the option of accepting or rejecting the offer. If the offer is rejected, both players receive nothing. If the receiver accepts the offer, both parties receive the division offered by the first player. Receivers' acceptance level is commonly interpreted as an indicator of the norm of fairness present in a society [6].

To explicitly test whether group leaders are expected to be more generous toward group members, study participants were randomly assigned to one of two variants of a UG (see section 4 in File S1 for the experimental scripts). In both variants, members of the farmer cooperatives acted as receivers, whereas in one treatment group the decider was another group member while in the second treatment group the decider was one of the group leaders. Comparing receivers' minimum acceptance levels between treatment groups allows us to assess whether leaders are subject to higher expectations.

Results from the UG suggest that group members hold their leaders to a higher standard: the minimum acceptance offer is significantly higher when farmers were paired with group leaders compared to other regular members. Group members will accept, on average, a minimum offer of 3.75 MUs from other group members, while they expect leaders to allocate at least 4.34 MUs (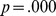).

## Discussion

In their seminal work on fairness in 15 diverse societies, Henrich et al. [31] find greater levels of prosocial behavior in large-scale, market integrated societies in which strangers regularly engage in mutually beneficial transactions than in small-scale societies based on kinship networks. Similarly, inter- and intra-cultural variations in levels of prosocial behavior have been explained on the basis of the patterns of social relationships between individuals [22]–[24]. In this paper we have shed some light on two basic mechanisms that support prosocial behavior in complex societies: group attachment and social position. Processes of categorization and generalization that induce individuals to regard the welfare and preferences of those beyond the immediate circle of kin and acquaintances trigger both mechanisms.

In more general terms, we argue that social organization, more than socio-emographic characteristics [8], [11], [19], determines one's other-regarding preferences. Most individuals are neither universally generous nor selfish. Instead, their willingness to share resources with others varies according to their relative position in the social structure.

In complex societies, individuals lie at the intersection of multiple social groups and identities and occupy different positions in the social structure. Our first key finding is that group attachment affects prosocial behavior above and beyond the level of proximity with group members, and that this attachment varies across groups. The strength of one's identification with a group affects the way in which individuals balance individual and collective interests. From this we conclude that social preferences, namely the extent to which individuals take the welfare of others into account, are group-specific. This finding qualifies classic social preference theories such as the ones developed by Fehr and Schmidt [61], or by Charness and Rabin [62].

Our second key finding is that unlike informal network positions, formal leadership positions are consequential for prosocial behavior. Sharing resources with in-group members is only weakly related to one's overall level of generalized altruism: individuals who occupy formal positions in the social structure do not give more to strangers and they do not give more to members of overlapping groups in which they do not hold leadership positions. By contrast, in-group favoritism is an emergent property of social organization, which inheres in the role individuals occupy in the social structure.

## Materials and Methods

All subjects consented to the data collection. IRB approval from Princeton University (protocol number 0000004287) has been obtained. Participants provided verbal consent in their own language to local survey interviewers. We opted for verbal consent because 30% of the subjects were illiterate, thus could not read the content of a written consent form. Moreover, there is a lot of skepticism among rural Ugandans toward signing papers presented by strangers, out of fear that someone might take away their land. The Ethic Committee at Princeton approved the consent procedure.

To increase the external validity of our findings we conducted our experiments with members of farmer groups that were created as part of one of Uganda's largest recent rural development interventions: the Agriculture Productivity Enhancement Project (APEP). Our “lab-in-the-field” experiments took place in 50 different groups in 9 districts across Uganda. In each location, around 30 farmers were randomly sampled from a list of all members of a local farmer cooperative, for a total of 1,541 subjects. We used a stratified, random, multistage cluster design to select our sample. In addition, we also included in our study all group members occupying a formal position of authority in their farmer group, which leads to a total sample of 2,597 subjects (see Section 2 in File S1 for further details on the sampling strategy).

In each of the 50 farmer organizations, we collected complete network information on the relationships among members of the farmer association council. Namely, each respondent was presented with a complete list of other council members' names, and, for each of them, was asked about their friendship – “Is [NAME] a close friend or do you just know him or her? By close friend, I mean that you (a) eat together regularly; (b) you can leave your child with him or her if you need to travel for several days; and (c) he or she will help you in case of a family death.” –, communication – “Do you have [NAME] 's phone number? ” –, and advice relationship –“In the past 12 months, have you asked [NAME] for information or advice on matters related to the farmer association? ”. Affirmative answers to these questions were recorded as a tie in the friendship, communication, and advice network, respectively.

Data were collected between July 2009 and September 2009 by a group of 60 experienced local interviewers, divided into three “language” teams. The scripts of the experiments were translated and back-translated from English into each of the native languages (Basoga, Luganda, and Ranyankole), and several pilot tests and debriefing sessions were conducted. Interviewers went through a two-week training period in classroom and field settings, which included training on human subjects issues as well as survey techniques, and they were supervised by team leaders throughout the entire data collection.

Our subjects received a participation fee, travel reimbursement, and their gains from the behavioral games played during the day. Each endowment in our dictator and ultimatum games was 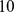 coins of 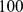 Ugandan Shillings (UGX), which are equivalent to half a day's wage in rural Uganda. Participants were payed the payoff of only one allocation decision in the DG, which was randomly selected after the game had been played.

## Supporting Information

File S1 **Supporting Information.**
(PDF)
