# Supplementary material for: The Effect of Group Attachment and Social Position on Prosocial Behavior. Evidence from Lab-in-the-Field Experiments
Source: PLoS One. 2013 Mar 26;8(3):e58750. doi: 10.1371/journal.pone.0058750 (PMC3608652; doi:10.1371/journal.pone.0058750)
Supplement: File S1 — Supporting Information. (PDF) [file pone.0058750.s001.pdf]

SUPPORTING INFORMATION
The Effect of Group Attachment and Social Position on Prosocial Behavior. Evidence from Lab-in-the-Field Experiments

Delia Baldassarri,[1*] Guy Grossman[2]

[1]New York University, Department of Sociology; delia.b@nyu.edu
[2]University of Pennsylvania, Department of Political Science

Contents

1 Supporting Analysis **2**
1.1 Descriptive Statistics . . . 2
1.2 Contribution by Type of Recipient: Test of the Social Standing Hypothesis . . . 3
1.3 Network Centrality and Prosocial Behavior . . . 4

2 Sampling Strategy **5**
2.1 Implementation . . . 7
2.2 Measurement of Key Variables . . . 7
2.3 Randomization Test . . . 8

3 Script of the Dictator Game **9**
3.1 Materials . . . 9
3.2 Introduction to the Activity . . . 9
3.3 Game Description and Instructions . . . 9
3.4 One-on-one Meeting . . . 11

4 Script of the Ultimatum Game **13**
4.1 Materials . . . 13
4.2 Setup . . . 13
4.3 Group Instructions . . . 13
4.4 One-on-one Meeting . . . 14

1 Supporting Analysis

1.1 Descriptive Statistics

	mean	sd	min	max
DG contribution: group member	4.07	1.99	0.00	10.00
DG contribution: co-villager	3.07	2.00	0.00	10.00
DG contribution: stranger	2.84	1.98	0.00	10.00
Farmer group leader	0.06	0.25	0.00	1.00
Village leader	0.25	0.43	0.00	1.00
Male	0.73	0.44	0.00	1.00
Age (units of 10)	4.61	1.34	1.40	9.50
Church attendance	2.99	0.52	1.00	4.00
Born in village	0.51	0.50	0.00	1.00
Education (Std.)	0.00	1.00	-2.80	1.95
Wealth (Std.)	0.00	1.00	-2.39	9.15

Table S1: Descriptive sample statistics. N=2,597.

1.2 Contribution by Type of Recipient: Test of the Social Standing Hypothesis

	Stranger (A)	Villager (B)	Group Member (C)
Village leader	0.101	0.235*	0.044
	(0.088)	(0.132)	(0.116)
Farmer group leader	0.017	0.166	0.400**
	(0.116)	(0.196)	(0.156)
Male	-0.004	-0.117	0.264*
	(0.101)	(0.148)	(0.136)
Age (units of 10)	0.033	0.046	-0.038
	(0.032)	(0.047)	(0.044)
Born in the village	-0.059	0.113	-0.020
	(0.090)	(0.134)	(0.118)
Church attendance	-0.072	-0.084	0.112
	(0.078)	(0.111)	(0.107)
Education (Std.)	-0.017	0.045	-0.033
	(0.044)	(0.065)	(0.059)
Wealth (Std.)	0.020	-0.050	0.053
	(0.044)	(0.065)	(0.058)
Intercept	2.868***	2.863***	3.139***
	(0.332)	(0.493)	(0.457)
$\sqrt{\psi_{(a)}}$	-1.127***	-2.115	-0.887***
	(0.275)	(3.922)	(0.281)
$\sqrt{\psi_{(b)}}$ -0.222***	-0.059	-0.175*	
	(0.078)	(0.109)	(0.102)
σ_e	0.580***	0.578***	0.554***
	(0.016)	(0.024)	(0.023)
N	2247	1042	1205
Log Likelihood	-4650	-2164	-2477

Standard errors in parentheses. * $p < 0.05$ ** $p < 0.01$ *** $p < 0.001$

Table S2: Average contribution to a stranger, co-villager, and farmer group member in the DG controlling for whether subjects hold formal leadership position in the village and farmer group. DV: contribution to stranger (A), village (B), or farmer group (C) member. Table reports results from a series of three-level random intercept linear regression models, in which individuals are nested within producer organizations and interviewers, in order to control for group and interviewer effects. $\sqrt{\psi_{(a)}}$ refers to variability between farmer groups, $\sqrt{\psi_{(b)}}$ refers to between interviewers variability, and σ_e is the estimated standard deviation of the overall error term.

1.3 Network Centrality and Prosocial Behavior

**Panel A: Friendship Network**			
	A: Degree	B: Betweenness	C: Eigenvector
Network Centrality	-0.008	0.000	0.013
	(0.01)	(0.00)	(0.38)
Intercept	4.458***	4.289***	4.281***
	(0.23)	(0.17)	(0.29)
$\sqrt{\psi_{(a)}}$	-0.097	-0.060	-0.058
	(0.15)	(0.14)	(0.15)
σ_e	0.712***	0.710***	0.710***
	(0.03)	(0.03)	(0.03)
Log Likelihood	-1305	-1306	-1306
**Panel B: Communication Network**			
	A: Degree	B: Betweenness	C: Eigenvector
Network Centrality	-0.001	-0.001	-0.054
	(0.01)	(0.00)	(0.47)
Intercept	4.319***	4.299***	4.330***
	(0.30)	(0.17)	(0.39)
$\sqrt{\psi_{(a)}}$	-0.062	-0.057	-0.062
	(0.14)	(0.14)	(0.14)
σ_e	0.711***	0.710***	0.711***
	(0.03)	(0.03)	(0.03)
Log Likelihood	-1306	-1306	-1306
**Panel C: Advice Network**			
	A: Degree	B: Betweenness	C: Eigenvector
Network Centrality	-0.001	-0.001	-0.054
	(0.01)	(0.00)	(0.47)
Intercept	4.319***	4.299***	4.330***
	(0.30)	(0.17)	(0.39)
$\sqrt{\psi_{(a)}}$	-0.062	-0.057	-0.062
	(0.14)	(0.14)	(0.14)
σ_e	0.711***	0.710***	0.711***
	(0.03)	(0.03)	(0.03)
Log Likelihood	-1306	-1306	-1306
N	599	599	599

Standard errors in parentheses. * $p < 0.05$ ** $p < 0.01$ *** $p < 0.001$

Table S3: Network Centrality and Prosocial Behavior. Results from multilevel random intercept linear regression models. Dependent variable: contribution to farmer group co-member in the DG. In all models, the key independent variable is informal position in one of three types of networks (friendship, communication and advice), measured using (A) degree, (B) betweenness, and (C) eigenvector centrality. To control for group effects, subjects are nested within farmer associations. $\sqrt{\psi_{(a)}}$ refers to variability between farmer groups, and σ_e is the estimated standard deviation of the overall error term.

2 Sampling Strategy

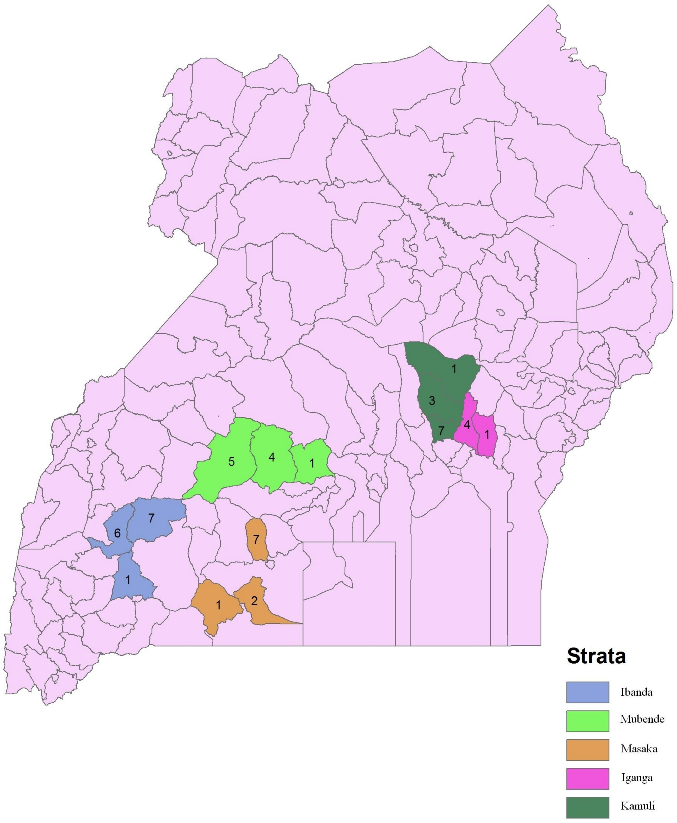

We followed a stratified, multistage cluster design. Starting from 5 district-areas (strata), we sampled 50 parish-level farmer associations, then 6 village-level farmer groups and 36 farmers from each farmer association. We followed a five-step sampling strategy, summarized in the table below.

Step	Sampling Unit (SU)	Number of SUs	Sampling Method
1	Target Population	105 DCs	Coffee growers
2	District-area	5 strata	Stratified – proportional to # of DCs in strata
3	Farmer Associations	50	unequal probability without replacement
4	Producer Organizations (POs)	6 per DC	Clustered – simple random sample.
5a	Group members	36 per DC	Clustered – probab proportional to POs size.
5b	Farmer Leaders	20 per DC	Complete population.

Step 1: Target Population. In order to reduce variability due to environmental factors, we limited our sample to farmer associations (DCs) that cultivate the same crop. Thus we included only coffee groups, as this was the most common crop. This decision reduced the universe of cases from 204 to 113 associations. An additional 8 DCs were excluded before sampling due to the following reasons: we excluded 2 associations from the Bugiri district because coffee is a

very unusual crop in that district. We further excluded 5 associations from the Busheni district because those groups were formed many years before APEP. Finally, we excluded from our sample a DC from the Kamwenge because it was the single DC in that district and surveying it would have been logistically complicated and prohibitively expensive. Our final universe comprises 105 coffee growing farmer associations, which were all founded after 2005.

Step 2: Strata. Based on power calculations performed on simulated data, we selected 50 associations, using a stratified random sample. Though our universe of farmer associations is spread over 9 districts, we grouped associations into 5 strata. Strata were defined by meaningful district-areas: neighboring districts that were covered by the same project field trainers and trading partners, and that share a dominant ethnicity and/or were historically part of the same district were grouped in the same strata (Fig. above).

Step 3: Farmer Associations. We draw independent probability samples of farmer associations from each stratum. We used unequal probability sampling without replacement to sample associations within strata (proportional to their size). The number of associations that were sampled from each stratum was proportional to the number of associations in each stratum. According to this scheme, sampled associations are representative at the stratum level.

Step 4: Village-level Farmer Groups. We want to be able to detect variation not only between associations but also within associations; i.e. in the success of village-level farmer groups that make-up each association. Thus, prior to sampling group members, we randomly sampled six farmer groups from each association. In the case that a farmer association had six or fewer village-level groups, we selected all of them.

Step 5a: Random Sampling of Group Members. The same number of respondents (36) was sampled from each farmer association. The number of sampled members from each of the six *sampled* village-level groups was proportional to the size of the groups. This assured that the sample is self-weighted. Total sample size is thus 50 DCs $\times$ 6 groups $\times$ 6 members per group = 1,800. We succeeded in surveying 1,784 out of the 1,800 sampled group members (99% response rate), out of which 1,541 participated in the public goods games (86% of the subjects sampled).

Step 5b: Complete Population of Farmer Leaders. We also included in our study all members occupying a formal position of authority in their farmer association. In order to collect basic information and complete network data on the DC leadership, from each DC we invited all the DC executives (i.e., manager, chairmen, secretary and treasurer) whether or not their PO was sampled, and (b) one or two PO representatives from each PO, *whether or not their PO was sampled.* To keep the number of representatives manageable, we adopted the following rule: when the number of POs that make the DC was up to 10, we invited two representatives from each PO (apart from the PO chair). When the number of POs was 11 and above, we invited only the most active representative. For example, when a DC had 8 POs, we invited 28 leaders based on their position (4 executives + 2*8 PO representatives +8 PO chairmen). In order to identify the "most active" representative, we invited the the PO representative that came to the largest number of meetings at the DC level in the past six months.

2.1 Implementation

Data were collected between July 2009 and September 2009 by a group of 60 experienced local interviewers. Interviewers, who were hired directly by the PIs and their project manager, were divided into three "language" teams. The eastern team covered 16 farmer associations in the Iganga and Kamuli districts, where Basoga is the local language. The central team covered 20 DCs from the Mubende, Mityana, Masaka and Rakai districts, where locals speak Luganda. Finally, the western team covered 14 DCs from the Kiruhura, Mbarara and Ibanda districts, where Ranyankole is the lingua franca. Interviewers went through a lengthly training in class (4 days) and in the field setting (4 days), which included training on human subjects issues as well as survey techniques. Interviewers were supervised by team leaders: on average, there was a team leader for every 5 interviewers. This made it possible to constantly screen the work of the interviewers and greatly increase the quality of our data.

In each sampled association, data was collected in four rounds. First, an interviewer scheduled a meeting with the executives of the farmer association. In that meeting the interviewer introduced the study to the leaders and asked for their cooperation. In that meeting the interviewer also administered the association-level questionnaire, as well as obtained a list of all DC council members. On the second day of interviews, the research team conducted interviews with group representatives to the DC council and with the chairmen of all village-level groups. All subjects were mobilized by the association's executives to a central location. In addition to the individual-level interviews, association leaders from each sampled village-group were asked to respond to a group-level questionnaire, and to provide a complete list of group members. Between the second and third days of interviews, the research team sampled 36 members from each sampled association (including 8 replacements). Immediately after the sampling procedure (see above), an interviewer travelled back to meet with the association leaders to hand them the list of sampled members and coordinate with them the next round of interviews. Once again, we relied on the associations leaders to mobilize the sampled members to a centralized location. On the third day of interviews, individual-level interviews were conducted with the sampled members and with representatives who were not present on the previous day. On that day we also conducted a set of behavioral games with both "ordinary" members and group leaders. Finally, the survey team traveled to each association for an additional day to interview sampled members or representatives who, for any reason, were not present in the previous days of interview.

2.2 Measurement of Key Variables

To construct a measure of each individual's education, we used information on respondents' literacy level, educational attainment, and English proficiency, as English is the lingua franca of the business and political class. Those variables were then combined into a summary index using principal component analysis. The wealth measure was constructed using questions about ownership of 12 assets, such as bicycles and livestock, which reflect the purchasing power of farmers in rural Uganda.

2.3 Randomization Test

Randomization Check using Survey Data

	Range		Sample	Villager	Member	OLS	
	min	max	mean	variant	variant	P-value	n
**Panel A: Demographics**							
Male	0	1	0.71	0.71	0.72	0.564	2020
Age	14	95	45.8	46.2	45.4	0.241	2020
Church attendance	1	4	2.99	2.98	2.99	0.820	2020
Can Write	0	1	0.77	0.78	0.75	0.157	2020
Advanced English	0	1	0.27	0.26	0.28	0.242	2020
Education attainment	0	6	2.01	1.99	2.02	0.522	2000
Education index (standardized)[a]	-2.79	1.95	.043	0.006	0.092	0.062	2000
Wealth index (standardized)[b]	-2.39	9.15	.024	-.018	.080	0.065	2015
Household head	0	1	0.81	0.84	0.84	0.996	2020
Total land (acres)	0.184	700	9.3	8.9	9.8	0.589	1940
**Panel B: Group Participation**							
Percent yield sold via group	0	1	0.54	0.73	0.55	0.494	1924
Years since joining farmer group	1	8	3.88	3.89	3.88	0.909	2012
N. group meeting attended past year	0	24	3.34	3.39	3.28	0.350	2013
Attended general assembly	0	1	0.67	0.68	0.65	0.107	2014
**Panel C: Agriculture practices**							
Plant seedlings past season	0	1	0.72	0.72	0.72	0.993	2011
Use fertilizers in past three years	0	1	0.18	0.17	0.18	0.330	2012
Use Herbicides in past year	0	1	0.31	0.30	0.31	0.610	2012
Hired labor to work on farm	0	1	0.34	0.34	0.35	0.889	2009

(a) The standardized summary index of education was constructed via principal component analysis using information on respondents' literacy level, educational attainment, and English proficiency, as English is the lingua franca of the business and political class.

(b) The standardized summary index of wealth was constructed via principal component analysis using questions about ownership of 12 assets, such as bicycles and livestock, which reflect farmers' purchasing power. Using asset ownership to measure the welfare of poor households is a commonly used technique in poor developing countries where monetary measures of income and wealth are problematic FilmerPritchett2001. For each asset, respondents were asked to provide information on the number of items they currently have.

3 Script of the Dictator Game

3.1 Materials

Each enumerator should have:
- A large cardboard privacy screen
- 20 100USH coins
- 1 Stranger board
- 1 PO member board
- 1 set Payment cards (1 Stranger, 1 PO)
- 1 set Activity 1 Game 1 Group 1 Record sheet

3.2 Introduction to the Activity

Thank you all for participating to this activity. My name is [enumerator 1] and this is my colleague [enumerator 2]. In this activity we will be playing with real money. You should know that whatever money you win in this and the next activities will be yours to keep and take home. This money is for your individual use, goes to your private pocket, and you can spend it as you want. You will receive the money from this activity, as well as your compensation, at the end of the day. The money comes from two American Universities, Princeton and Columbia. You should understand that this money does not come from our own (private) pocket. It is money given to the research team by the University to use for research.

Before we begin, I want to tell you the rules that we must follow. I am about to explain the first activity and it is important that you listen as carefully as possible, because only people who understand the activity will actually be able to participate. We will run through some examples here while we are all together. While in the group, you cannot ask questions or talk. This is very important. Please be sure that you obey this rule, because it is possible for one person to spoil the activity for everyone. If one person talks about the activity while sitting in the group or with other people later, this person will not be allowed to participate to the activity today. Do not worry if you do not completely understand the activity as we go through the examples here in the group. Each of you will have a chance to ask questions in private to be sure that you understand.

Finally, you will be playing in groups of two individuals, but you would not be told the name of the person and you would not be able to see the person you are playing with. Similarly, we would not reveal your name to the person you are playing with or anybody else. Only us, the research team, will know about your decisions, and well register them using an identification code, and not your name.

3.3 Game Description and Instructions

This first activity involves pairs of individuals. Each pair will be given a total of ten 100 USH coins, totaling 1,000 USH. The First participant, the Decider, has to decide how to allocate the money between her/himself and the Second participant, the Receiver. The Decider will take

home whatever he has decided to allocate to himself, and the Receiver will take home whatever he has been allocated by the Decider.

All the people in this room have been chosen to participate as Deciders. As a Decider, you have to decide how to split the 1,000 USH between you and a Receiver. Here are 4 examples of how you could split the money:

Demonstrate on "Generic" Board

- if you give 1,000 USH (10 coins) to the Receiver, the Receiver will receive 1,000 USH and you will walk away with nothing at the end of the day.
- if you give the Receiver 200USH (2 coins), s/he will receive 200 USH and you will walk away with 800USH (8 coins).
- if you give the Receiver 700 USH (7 coins), s/he will receive 700 USH and you will walk away with 300 USH, (3 coins).
- if you give the Receiver 0 coins, s/he will receive nothing and you will walk away with 1,000 USH (10 coins).

We will ask you to make a decision two times for two different individuals. In the first case youll be asked to be the Decider and allocate money between you and a Stranger. You will use this board [pull out stranger board and set up 10 coins.] After you make your decision, a stranger will receive a box with the money. The Receiver will not be told your name or given any information about you. This stranger is a person who you do not. We have chosen this person in the following way: from the list of all the people in your sub-county, we have randomly selected a few people. The person that will be receiving is one of them. We will show you the picture of some of them, to make sure that you have never met any of them.

The second time, you will be playing again as a Decider, but this time the Receiver will be a member of your PO. You will use this board [pull out PO-member board and set up 10 coins.] After you make your decision, a member of your PO will receive a box with the money. The Receiver will not be told your name or given any information about you. He will be told only that the money comes from a co-PO member. This person might be here today or he might not be here. The only thing you know is that he is a member of your PO.

You will meet with the enumerators one-on-one. After you have decided how to allocate the money in each situation, we will determine which activity you will be paid for. Youll have to pick between two cards. One card has the symbol of the Stranger on it, while the other has the symbol of the co-PO member on it [show cards]. You will pick one of the cards, and you will take home the money you have made in the activity that you have randomly selected. Be careful, since you do not know in advance for which activity you are going to be paid for, you have always to decide as if you were dividing real money.

Before making your decision, the enumerator will explain the activity again, and let you ask any question you might have. While waiting during the one-on-one meetings with enumerators, no one should talk about the activity or their decisions. If you have a question, please ask the enumerator, not another participant. Also, you are not allowed to speak about the activity with

anybody during the day. You can talk about it only on your way home, after all the activities are over.

Now we are going to call each of you one-by-one to meet with an enumerator.

3.4 One-on-one Meeting

Each booth should be set up before the participant comes for their one-on-one interview. The boards with the two symbols should be placed in front of the participant with ten 100USH coins on each board on the"Personal Pocket side. Record the participants ID number in the "ID number Column. If you are Enumerator 2, first describe the co-PO member, and second, the stranger. Report the results in the appropriate column of the Recording sheet.

You have been selected to be a Decider. You have been given a total of ten 100USH coins, totaling 1,000USH, and have to decide how to allocate the money between you and the Receiver. You will make both decisions when I have finished explaining the activity.

Here you are dividing the money between yourself and a Stranger [Point to the corresponding board]. The Stranger is someone from your sub-county who has been selected at random from a complete list of all the members of the sub-county. Here some pictures of people in your sub-county who have been selected to participate with us. The person you are giving to is one of them. This person will NOT be told your name or given any information about you.

Here you are dividing the money between yourself and a member of your PO [Point to the corresponding board]. This person will NOT be told your name or given any information about you. He will not be told only that the money comes from a co-PO member. This person might be here today or not. The only thing you know is that he is a member of your PO.

In both cases, you will take home whatever you have decided to allocate to yourself, while the Receiver will take home whatever you have decided to give him/her. You do not have to give any money to anyone if you do not want to. Remember that you are dividing real money, and you will be paid for one of these decisions.

Lets start with an example. If you decide to give a person 300USH (3 coins), then you will have 700USH (7 coins) for yourself.

Do you have any question for me?

Let me now ask you a question: If you give 600USH (6 coins) to a person, how many do you keep to yourself?

If the participant responds correctly, go ahead with the Game! If not, give the answer, and ask another question:

If you give 200 USH (2 coins), how much are you keeping for yourself?

If the participant responds correctly, go ahead with the Game! If not, give the answer, explain, but put an ' next to the participants decision.*

You now have a few minutes to make their decisions for all three situations. When you are finished, please let me know.

Record the number of coins the participant has given to the Stranger in the"Offer - Stranger Column, and the number of coins the participant has given to the co-PO member in the"Offer co-PO Column.

Show the participant the 2 payment option cards.

Now we will decide which situation you will be paid for. I will shuffle these two cards, and you will pick one. The card you pick will indicate which decision you will be paid for.

it Shuffle cards, have participant pick one. Record under the Pick Column if the card is a Stranger, or a PO Member.

Refer to the appropriate board and point out how much the participant kept in the selected activity. Write the number under PAYMENT column.

In the situation for the card you picked, you kept [number from PAYMENT column], so that is how much you have made from this activity. At the end of the day you will be given [number] USH in addition to the participation fee.

Relocate the 20 coins back to the starting positions.

Thank you. You can now go. Please, call person [ID number]. (alternatively, call the person yourself)

Repeat with everyone in the group.

4 Script of the Ultimatum Game

4.1 Materials

Each enumerator should have:
- A large privacy screen
- 10 100 USH coins
- 1 “co-PO/Personal Pocket” board
- Sheets for recording results and pen

4.2 Setup

Group will sit together while instructions for the game and examples are introduced. Subjects have been already divided and randomly assigned in the previous game. Two enumerators will each have a private area/booth set up close by. Within the booth, there should be an area with ten USH 100 coins placed on a “co-PO/Personal Pocket” board. Coins placed on the Personal Pocket side are for the Decider, coins placed on the co-PO are for the Receiver, who is an anonymous member of the farmer group.

4.3 Group Instructions

You are now going to face a new set of decisions. This is NOT the same decision as before, so please listen very carefully.

This activity involves pairs of individuals. Both persons are PO members. Each pair will be given a total of ten 100 USH coins, totaling 1,000 USH. The first participant, the Decider, has to decide how to allocate the money between her/himself and the second participant, the Receiver. The Receiver can either accept the offer or reject it. If s/he accepts the offer, the Receiver will walk away with what s/he has been given by the Decider, and the Decider will walk away with what s/he has kept. If the Receiver rejects the offer, both participants will walk away with nothing.

Half of you will be selected to be Deciders, the other half will be Receivers. This selection is made randomly by chance. Also, at the end of the day you will be paired up randomly.

Here is an example of how the transaction can go.

Demonstrate on the board, using actual coins.

Imagine that the Decider offered 200 USH to the Receiver and kept 800 USH for him/herself. If the Receiver accepts the offer, he will receive 200 USH and the Decider will receive 800 USH. If the Receiver rejects the offer, both the Decider and the Receiver will walk away with nothing.

Now we are going to call each of you one-by-one to meet with an enumerator. While waiting during the one-on-one meetings with enumerators, no one should talk about the game and their

decisions. In making your decision, remember that you are deciding on the allocation of real money, that will be given you at the end of the day.

4.4 One-on-one Meeting

Enumerator 1 - Receivers

Each booth should be set up before the member comes for their one-on-one interview. The large, divided card should be placed where the player will sit, with the 10 coins on the Private Pocket side.

Record the players ID number in the ID Column.

`NOTE: In version 2 of the game the Decider is a PO-leader`

You and another PO member have been allocated 1,000 USH. You have been selected to be a Receiver. The other PO-member is now deciding how many coins he would like to give to you. You may accept or reject his or her offer. If you accept the offer, s/he will walk away with what he has kept, and you will walk away with what s/he has offered to you. If you reject the offer, s/he will walk away with nothing, and you will walk away with nothing.

1. Lets start with an example. You have been offered 400 USH by the PO-member. If you accept the offer, you get 400 USH and the PO-member gets 6 USH. If you reject the offer, both you and the Decider will get nothing.

Now, Im going to ask you a question. If you decide to reject the offer, how much do you get? And how much does the other PO-member get?

If Player answers correctly, move on to the game. If not, ask another question. (Answer should be 0,0)

2. Lets try a different question. You have been offered six coins (600USH) by the PO-member. You accept the offer. How many coins do you get? How many coins does the other PO-member get?

If player answers correctly, move on. If not, place an asterisk next to their ID number, but play anyway. (answer should be 6,4)

Now I will go over a list of possible offers the other PO-member could be making so that we know which offers you would accept and which offers you would reject. Remember that the Decider knows that you are a PO member, but does not know your name. Remember if you accept the offer, you will walk away with what you have been offered,

and the PO-member will walk away with what he has kept. If you reject the offer, you will both walk away with nothing.

Ask the player each of the following questions. For each one, move the coins between the spaces on the board to create a visual demonstration.

Mark a 1 on the response sheet if the individual would accept an offer of that many coins; mark a 0 if they indicate they would reject such an offer.

If a PO member offered you 10 coins (1,000 USH) and kept 0 for himself, would you accept or reject?
If the offer is rejected, visually represent it by taking away all the coins
If a PO member offered you 9 coins and kept 1 for himself, would you accept or reject?
If the offer is rejected, visually represent it by taking away all the coins
If a PO member offered you 8 coins and kept 2 for himself, would you accept or reject?
If the offer is rejected, visually represent it by taking away all the coins
If a PO member offered you 7 coins and kept 3 for himself, would you accept or reject?
If the offer is rejected, visually represent it by taking away all the coins
If a PO member offered you 6 coins and kept 4 for himself, would you accept or reject?
If the offer is rejected, visually represent it by taking away all the coins
If a PO member offered you 5 coins and kept 5 for himself, would you accept or reject?
If the offer is rejected, visually represent it by taking away all the coins
If a PO member offered you 4 coins and kept 6 for himself, would you accept or reject?
If the offer is rejected, visually represent it by taking away all the coins
If a PO member offered you 3 coins and kept 7 for himself, would you accept or reject?
If the offer is rejected, visually represent it by taking away all the coins
If a PO member offered you 2 coins and kept 8 for himself, would you accept or reject?
If the offer is rejected, visually represent it by taking away all the coins
If a PO member offered you 1 coins and kept 9 for himself, would you accept or reject?
If the offer is rejected, visually represent it by taking away all the coins
If a PO member offered you 0 coins and kept 10 for himself, would you accept or reject?
If the offer is rejected, visually represent it by taking away all the coins

Now that we know when you would accept or reject, we will match it with the offer made by the other PO member. Once we know the offer, we will know how much you will be paid, and this will be given to you at the end of the day. Thank you. You may sit down.

Enumerator 2 - Deciders

Each booth should be set up before the member comes for their one-on-one interview. The large, divided card should be placed where the player will sit, with the 10 coins on the Private Pocket side.

Record the players ID number in the ID Column.

You and another PO member have been allocated 1,000 USH. You have been selected to be the Decider, and have to decide how to divide the money between you and the Receiver. If the receiver accepts your offer, you will walk away with what you have kept, and s/he will walk away with what you have offered him/her. If s/he rejects the offer, s/he will walk away with nothing, and you will walk away with nothing.

1. Lets start with an example. If you offer 400 USH to the other PO member and this person accepts the offer, you get 600 USH and the other PO member gets 400 USH.

Now, Im going to ask you a question to see if you understand. If your offer is rejected, how many USH do you get? And how many USH does the other PO member get?

If Player answers correctly, move on to the game. If not, ask another question. (Answer should be 0,0)

Lets try a different question. You offered 600 USH to the other PO member and he accepts the offer. How many USH do you get? How many USH does the other PO member get?

If player answers correctly, move on. If not, place an asterisk next to their ID number, but play anyway. (answer should be 6,4)

Now it is time to make your decision. Remember that the other PO member knows that you are a PO member, but would not be told your name. All the coins are currently in the space for you. Move however many coins you want to offer to the other PO member to the other side of the board. I will give you a few moments to make your decision. Let me know when you are finished.

Record their response in the Offer Column. Return the coins to their starting position.

I will offer the amount you have given to a PO member. If he accepts it, you will receive this amount (point to the money he has kept) at the end of the day. If he rejects it, you will receive nothing. You will be paid at the end of the day.

Thank you. You may sit down.
